# The Role of Oxidative Stress in Cardiac Disease: From Physiological Response to Injury Factor

**DOI:** 10.1155/2020/5732956

**Published:** 2020-05-14

**Authors:** Rossella D'Oria, Rossella Schipani, Anna Leonardini, Annalisa Natalicchio, Sebastio Perrini, Angelo Cignarelli, Luigi Laviola, Francesco Giorgino

**Affiliations:** Department of Emergency and Organ Transplantation–Section of Internal Medicine, Endocrinology, Andrology and Metabolic Diseases, University of Bari Aldo Moro, Piazza Giulio Cesare, 11, I-70124 Bari, Italy

## Abstract

Reactive oxygen species (ROS) are highly reactive chemical species containing oxygen, controlled by both enzymatic and nonenzymatic antioxidant defense systems. In the heart, ROS play an important role in cell homeostasis, by modulating cell proliferation, differentiation, and excitation-contraction coupling. Oxidative stress occurs when ROS production exceeds the buffering capacity of the antioxidant defense systems, leading to cellular and molecular abnormalities, ultimately resulting in cardiac dysfunction. In this review, we will discuss the physiological sources of ROS in the heart, the mechanisms of oxidative stress-related myocardial injury, and the implications of experimental studies and clinical trials with antioxidant therapies in cardiovascular diseases.

## 1. Introduction

Reactive oxygen species (ROS) are highly reactive chemical species containing oxygen, including the superoxide (O_2_^−^) and the hydroxyl (OH^−^) anions, and hydrogen peroxide (H_2_O_2_). Under normal physiological conditions, ROS levels are strictly controlled through the activity of antioxidant enzymes, including superoxide dismutase, catalase, and glutathione peroxidase [1–3]. In the heart, ROS play a fundamental function in cell homeostasis when present at low concentrations, since they regulate multiple physiological signaling pathways and biological processes. Oxidative stress is defined as a dysregulation between the production of ROS and the endogenous antioxidant defense mechanisms, resulting in excessive ROS linked to multiple pathophysiological pathways in the heart. This review will summarize the current knowledge regarding ROS generation and their physiological and pathological actions in the heart. Specifically, the ability of ROS to regulate differentiation, proliferation, and excitation-contraction coupling in the heart under physiological condition and the involvement of ROS in multiple cardiac diseases under oxidative stress conditions will be evaluated. Additionally, the role of ROS under particular pathological conditions, such as chemotherapy-induced cardiotoxicity, atrial fibrillation, and diabetic cardiomyopathy, will be also discussed. Finally, we will focus on the current knowledge regarding clinical trials with antioxidant therapies in cardiovascular diseases related with oxidative stress.

## 2. ROS

### 2.1. ROS, Antioxidant Systems, and Cellular Sources of ROS in the Heart

ROS are oxygen-based chemical species characterized by high reactivity, physiologically generated in the cells as by-products of cellular metabolism or as toxic molecules involved in host defense [4–6]. They include free radicals, species with one or more unpaired electrons, such as superoxide (O_2_^−^) and hydroxyl (OH^−^) anions, and compounds such as hydrogen peroxide (H_2_O_2_), which can be converted to radicals, generating hydroxyl radicals via Fenton chemistry [7]. O_2_^−^ could both lead to the formation of other ROS, such as H_2_O_2_ and OH^−^, and combine with nitric oxide (NO) to form peroxynitrite (ONOO^−^) [8]. In addition, OH^−^ could arise from electron exchange between O_2_^−^ and H_2_O_2_ via the Haber-Weiss reaction [9]. ROS participate in both normal and pathological biochemical reactions. An excessive ROS concentration results in oxidation and damage to DNA, membranes, proteins, and other macromolecules. Specifically, the most studied cellular sources of ROS within the heart include cardiomyocytes, endothelial cells, and neutrophils [9]. Multiple antioxidant defense systems exist to counteract ROS accumulation by scavenging and converting ROS to nontoxic molecules. These systems are both enzymatic and nonenzymatic: enzymes include catalase, glutathione peroxidase (GSHPx), superoxide dismutase (SOD), and glutaredoxins (Grxs); nonenzymatic antioxidants include vitamins E and C, beta-carotene, ubiquinone, lipoic acid, urate, and reduced glutathione [7, 10, 11]. Reduced glutathione (GSH) is the main low-molecular-weight thiol-containing peptide present in most living cells and represents the most relevant natural antioxidant [11]. It acts as a scavenger of electrophilic and oxidant species either in a direct way or through enzymatic catalysis, since GSH is the cosubstrate of GSHPx and allows the reduction of peroxides and the production of GSSG [11].

SOD converts O_2_^−^ to H_2_O_2_, which is broken down by GSHPx and catalase to H_2_O. The GSHPx enzyme represents an important defense mechanism within the heart and is highly expressed especially in the cytosolic and mitochondrial compartments [12]. Glutaredoxins, whose major isoforms in mammals are Grx1, Grx2, and Grx5, are glutathione- (GSH-) dependent oxidoreductases with low molecular masses able to catalyze S-glutathionylation and deglutathionylation of proteins to protect SH groups from oxidation and restore functionally active thiols [13]. The thioredoxin (Trx) system represents an additional integrated antioxidant defense system, composed of NADPH, thioredoxin reductase (TrxR), and thioredoxin [14], and provides the electrons to thiol-dependent peroxidases (peroxiredoxins) to remove ROS. Peroxiredoxins (Prxs) are 20–30 kDa proteins, expressed as different isoforms and located in different cellular compartments. In addition to their peroxidase activity, they also act as molecular chaperones and phospholipase A2. Mammalian cells contain six Prxs, which are divided into three groups based on their structure and the catalytic mechanisms, and most Prxs function as homodimers, while the 2-Cys Prxs also form decamers [15].

### 2.2. Sources of ROS in Heart Cells

There are several potential sources of ROS in the heart, including mitochondria, xanthine oxidoreductase, nitric oxide synthases, NADPH oxidase, cytochrome P450, and monoamine oxidases (Table 1).

### 2.3. Mitochondrial ROS

Mitochondria produce energy, in the form of ATP, through a multistep process, which is represented by oxidative phosphorylation and electron flow across the electron transport chain (ETC), involving five protein complexes (complexes I-IV of the respiratory chain and the ATP-synthase complex) and two shuttles (coenzyme Q and cytochrome C) [16]. ROS generation in mitochondria is related to the partial reduction of O_2_ to O_2_^−^ by complexes I and III of the ETC. However, other proteins may also trigger mitochondrial oxidative stress, such as the p66^shc^ protein, a 66 kDa cytosolic protein encoded by the *Shc* gene that upon stress may translocate to mitochondria [17]. p66^shc^ accepts electrons from cytochrome C, and this results in the formation of H_2_O_2_ [18]. Several studies have demonstrated that targeting p66^shc^ could represent a potential strategy to reduce mitochondrial ROS generation [19–21]. For example, Rota et al. reported that in a model of insulin-dependent diabetes mellitus, the generation of ROS leads to senescence and apoptosis of cardiac progenitor cells (CPC), a specific class of cardiac stem cells [22]; ablation of the p66^shc^ gene prevents these negative adaptations of the CPC compartment, limiting the acquisition of the heart senescent phenotype and the development of heart failure in diabetes [22]. An intimate link has been demonstrated among ROS, mitochondrial DNA damage, and defects in the electron transport function, which might play an important role in the development and progression of left ventricular remodeling and failure in a murine model of myocardial infarction [23]. Complexes I and III are the best characterized enzyme complexes mediating ROS generation in the mitochondria and are responsible for the majority of mitochondrial ROS in cardiovascular physiology and disease [24]. Specifically, flavin mononucleotide and flavin mononucleotide-binding domain and ubisemiquinone and quinone-binding domain of complex I, as well as unstable semiquinone mediated by the Q cycle of complex III, control ROS production [24]. Moreover, oxidative posttranslational modification by glutathione in complex I and complex II affects enzymatic catalysis, protein-protein interactions, and enzyme-mediated ROS production [24]. ROS produced by ETC may also enhance oxidative stress in the mitochondria via induction of the prooxidant activity of aconitase, a Krebs cycle enzyme [24]. The effects of mitochondrial antioxidant modifications have also been studied. Indeed, TrxR2 plays a pivotal role in heart function, since the ventricular heart wall of TrxR2^−/−^ embryos is thinned, proliferation of their cardiomyocytes is reduced, and cardiac tissue-restricted ablation of TrxR2 results in fatal dilated cardiomyopathy [25]. Additionally, overexpression of catalase targeted to mitochondria in mice attenuates cardiac aging and protects from cardiac disease [26], whereas overexpression of mitochondrial peroxiredoxin-3 prevents left ventricular remodeling and failure after myocardial infarction in mice [27].

### 2.4. Xanthine Oxidoreductase

Xanthine oxidoreductase (XOR) represents another important major source of ROS in the human heart. It is a homodimer of 300 kDa, composed of the molybdopterin cofactor (Mo-Pt), two Fe-S centers, and a flavin adenine dinucleotide- (FAD-) containing domain [28, 29]. This enzyme is normally expressed as the dehydrogenase form (XDH) but under inflammatory conditions switches from the reductase form to the oxidase form (XO) through the oxidation of the cysteine residues 535 and 992 and/or proteolytic conversion [28, 30]. Both forms are responsible for the oxidation of xanthine to uric acid, thus promoting a flux of electrons that are used to reduce NAD^+^ to NADH, in the case of the XDH form, and molecular oxygen to H_2_O_2_ and O^2-^ in the case of the XO form. Multiple studies have demonstrated that XOR activity is regulated both at the gene expression and posttranslational levels [31]. Specifically, high oxygen tension, NO, and other products and by-products of XOR reactions, including O_2_^−^ [32], H_2_O_2_, and OH^−^ [33], have been implicated as negative regulators of XOR activity, whereas hypoxia [34] and cytokines, such as TNF-*α*, IFN-*γ*, IL-6, and IL-1, may activate XOR gene transcription [35]. It has been suggested that angiotensin II may promote endothelial oxidative stress by XO activation; conversely, in patients with coronary disease, losartan therapy reduces endothelium-bound XO activity, likely contributing to improved endothelial function [36]. Cappola et al. have demonstrated that short-term administration of allopurinol, a selective XO inhibitor, is able to improve myocardial efficiency in patients with idiopathic dilated cardiomyopathy, suggesting that XO may contribute to mechanoenergetic uncoupling in human heart failure. Furthermore, in failing heart, the XDH/XO protein expression is increased when compared with normal myocardium [37]. Thus, free radical production by XO may be an important cause of impaired myocardial energy utilization, and XO inhibition may provide a novel therapeutic strategy for the treatment of congestive heart failure [37].

Minhas et al. have supported the idea that XOR is the primary source of ROS generation in the failing heart and that its upregulation contributes to maladaptive cardiac hypertrophy, directly participating in the progression of LV failure, since chronic XO inhibition with oxypurinol reverses left ventricular (LV) remodeling and improves LV function following experimental myocardial infarction in rats [38]. Chronic XO inhibition also prevents myofibrillar protein oxidation and preserves cardiac function in a transgenic mouse model of cardiomyopathy [39].

### 2.5. Nitric Oxide Synthases

Nitric oxide synthases (NOSs) are a family of enzymes that catalyze the production of NO and citrulline from oxygen and L-arginine, as substrates. In this process, electrons are transferred from NADPH, bound to the C-terminal reductase domain, to the heme iron and cofactor tetrahydrobiopterin (BH_4_) in the N-terminal oxygenase domain. Three NOS isoforms are of great interest in myocardium: endothelial NOS (eNOS or NOS3), inducible NOS (iNOS or NOS2), and neuronal (nNOS or NOS1) [40]. eNOS is expressed in coronary arteries and endocardium endothelial cells, as well as in cardiomyocytes and cardiac conducting tissue [41]. eNOS exerts its main effects through the release of NO from nearby coronary microvascular endothelium. The paracrine effects of eNOS-derived NO are cGMP-dependent and include hastening relaxation and increasing myocardial distensibility, resulting in the inhibition of *β*-adrenergic inotropy and reduction of mitochondrial respiration. eNOS is also found in cardiomyocytes (mostly in caveolae), where it is involved in mediating the positive inotropic response to sustained stretch by increasing the ryanodine receptor (RyR) activation [41].

Myocardial nNOS is preferentially localized to the sarcoplasmic reticulum (SR). It has been suggested that nNOS-derived NO may inhibit Ca^2+^ influx through the L-type Ca^2+^ channels and stimulate SR Ca^2+^ reuptake by promoting phospholamban (PLN) phosphorylation. nNOS-derived NO may also modulate the inotropic response to *β*-adrenergic stimulation and inhibit XOR activity, thereby limiting myocardial oxidative stress and, indirectly, increasing NO availability within the myocardium [41]. In particular, Khan et al. have shown that nNOS and XOR are colocalized in the SR of murine cardiomyocytes and that absence or inhibition of nNOS is associated with an increased XOR-dependent superoxide generation, suggesting the possibility that the cardiac phenotype of nNOS^−/−^ mice, characterized by the presence of ventricular hypertrophy, may be a consequence of increased myocardial oxidative stress [42].

iNOS-derived NO is considered to have detrimental effects on the myocardium. Indeed, upregulation of iNOS by IL-1*β* and IFN-*γ* induces apoptosis in neonatal rat cardiomyocytes and cytokine- and peroxynitrite-induced apoptosis of cardiomyocytes is inhibited by treatment with a peroxynitrite scavenger [43]. Moreover, mice with myocardial iNOS overexpression showed cardiac fibrosis, cardiomyocyte death, cardiac hypertrophy, and cardiac dilatation [44].

BH_4_ depletion, because of its oxidation and/or reduced synthesis, can result in uncoupling of NOS [45]. Uncoupled NOS generates more ROS and less NO, shifting the nitrosoredox balance and leading to adverse consequences on the cardiovascular system. Thus, reduced BH_4_ and uncoupled NOS play an important role in I/R injury, cardiac hypertrophy, and remodeling [46]. Conversely, an increased NO bioavailability can be considered a universal mechanism for cardioprotection against these types of damage.

Specifically, under conditions of limitation of L-arginine or BH_4_ or in the presence of high-glucose concentration, eNOS becomes unstable (on protein gels, it appears more as monomer) and electrons become diverted to molecular oxygen rather than to L-arginine, resulting in O_2_^−^ formation and leading to eNOS uncoupling [47–49]. Moreover, ROS generated by eNOS can further oxidize BH_4_ to BH_2_, thus enhancing this condition [16].

Pressure overload triggers eNOS uncoupling, which in turn contributes to dilatory remodeling and cardiac dysfunction in mice. Reversal of this process by BH_4_ treatment suggests a potential treatment to ameliorate the pathophysiology of chronic pressure-induced hypertrophy [50]. The same authors have demonstrated that, in a transgenic eNOS knockout model with low ROS production, severely pressure-loaded hearts develop only modest concentric hypertrophy with limited fibrosis and without LV cavity dilation [50]. Moreover, hearts with increased ROS derived from uncoupled eNOS also show increased metalloproteinases activation, which, in turn, is responsible for degradation of extracellular matrix enhancing left ventricular dilatation and aggravating cardiac function [51].

### 2.6. NADPH Oxidases

The NADPH oxidases (Nox) are a family of seven membrane-bound enzymes and represent the major sources of ROS in the cardiovascular system [52–54]. They catalyze the reduction of molecular oxygen to O_2_^−^ by using NADPH as electron donor. Nox contains a catalytic unit that forms a heterodimer with a lower molecular weight subunit called p22^phox^ and 4 cytosolic regulatory subunits, p40^phox^, p47^phox^, p67^phox^, and the small GTP-binding protein Rac [55]. Among these, Nox2 is abundantly expressed in cardiomyocytes [56–58], endothelial cells [52], and fibroblasts [59–61]; Nox4 is expressed in endothelial cells [62], cardiomyocytes [56], and fibroblasts [59, 63]. Nox2 is a sarcolemmal enzyme that is activated by multiple stimuli, including angiotensin-II (Ang-II), endothelin-1 (ET-1), TNF-*α*, growth factors, cytokines, and mechanical forces [40, 64]. In contrast, Nox4 is found in intracellular membranes and is constitutively active [40, 65].

Nox activity increases in the failing heart [66]. Indeed, failing myocardium of patients with ischemic cardiomyopathy (ICM) or dilated cardiomyopathy (DCM) is characterized by upregulation of Nox-mediated ROS release and is associated with increased Rac1 activity. Conversely, statin treatment inhibits myocardial Rac1-GTPase activity [67]. Nox proteins are also involved in angiotensin-II-induced cardiac hypertrophy: it has been proposed that puerarin, an isoflavonoid, and polydatin, a resveratrol glucoside, have antioxidative and cardioprotective effects because they suppress angiotensin II-induced cardiac hypertrophy by inhibiting Nox-induced superoxide generation in murine cultured cardiomyocytes [68, 69]. Interestingly, ROS produced by Nox can promote further ROS generation by other sources. For example, O_2_^−^ from Nox may activate XOR [52, 70] and degrade BH_4_ leading to NOS uncoupling, as observed in diabetes and hypertension [71].

### 2.7. Cytochrome P450 Oxidase

The cytochrome P450 enzymes (CYPs) belong to a family of heme proteins that catalyze the metabolism of a great number of endogenous and exogenous substrates [72–74]. Cytochrome P450 2E1 (CYP2E1) is mainly located in the endoplasmic reticulum and is among the most active CYPs in producing ROS [75]. The expression level of CYP2E1 increases significantly in human heart tissues under ischemia [76]. Moreover, CYP2E1 is an important gene in the pathogenesis of dilated cardiomyopathy, since knockdown of CYP2E1 significantly ameliorates dilated and thin ventricles and dysfunctional contraction in cTnT^R141W^ transgenic mice, a model of dilated cardiomyopathy, by reducing oxidative stress, activation of caspase-3 and caspase-9, release of cytochrome c, and apoptosis in the myocardium [76].

### 2.8. Monoamine Oxidases (MAO)

Monoamine oxidases (MAO) are localized in the outer mitochondrial membrane and exist as two isoforms, MAO-A and MAO-B [77]. These enzymes are involved in the regulation of metabolism or degradation of catecholamines and other biogenic amines in mammals and are both expressed at equivalent levels in the human heart [78]. MAO use a FAD cofactor to catalyze the oxidative deamination of several monoamines, including neurotransmitters (e.g., serotonin, norepinephrine, and dopamine) and exogenous amines ingested with normal diets (tyramine), generating H_2_O_2_ and the corresponding aldehydes as by-products. It was shown that MAO expression and their ability to produce ROS increase with age [79] and in age-associated chronic diseases (i.e., hypertension, pressure overload, and diabetes) [80–83]. Additionally, MAO-A-induced oxidative stress triggers p53 activation, leading to downregulation of peroxisome proliferator-activated receptor-gamma coactivator-1*α* (PGC-1*α*), a master regulator of mitochondrial biogenesis; moreover, cardiomyocytes transfected with a MAO-A adenovirus in the presence of tyramine show impaired lysosome function and acidification, leading to autophagic flux blockade and altered mitochondrial quality control [84]. Likewise, genetic deletion of MAO-B protects against oxidative stress, apoptosis, and ventricular dysfunction in a model of pressure overload [85].

## 3. Physiological and Pathological Actions of ROS

ROS are involved in the so-called redox signaling pathways, in which they act as important effectors of multiple intracellular responses. However, the physiological or pathological role of ROS depends on their type, concentration, and site of production. At low levels, ROS are involved in physiological processes, including excitation-contraction coupling (ECC) and cell differentiation and proliferation. Conversely, when ROS levels are high, they can modify the molecular structure and function of intracellular molecules. For example, ROS have the ability to affect the integrity of genomic DNA by inducing mutations, to cause structural modifications to proteins through enzymatic alterations or inactivation, and to alter intracellular lipids through lipid peroxidation [40]. Additionally, O_2_^−^ may react with NO, leading to inactivation of NO and subsequent loss of NO effect and resulting in generation of peroxynitrite (ONOO^−^) species and endothelial dysfunction [52]. This reaction tends to occur both when O_2_^−^ and NO levels are high and when antioxidant activity is low. In the heart, all these deleterious events could trigger cardiomyocyte dysfunction and death through apoptosis and cause contractile dysfunction, impaired cardiac remodeling, fibrosis, hypertrophy, and heart failure.

### 3.1. Physiological Roles of Cardiac Redox Signaling Pathways

#### 3.1.1. Differentiation and Proliferation

The cellular redox balance is an important regulator of differentiation and proliferation in many cell types [86, 87], including the cardiomyocytes [88–90]. Indeed, both mechanical force and electrical stimulation increase the proportion of beating cardiomyocytes within embryo bodies, which in turn is associated with increased ROS intracellular levels [91–93]. Conversely, agents that scavenge or reduce ROS levels affect cardiomyocyte formation [92, 94]. It has been suggested that ROS generated by the Nox family of NADPH oxidases may act as second messengers regulating cell growth and differentiation. Specifically, downregulation of Nox4, the major Nox isoform expressed during the early stages of differentiation in embryonic stem cells, suppressed cardiomyocyte differentiation, and this was rescued by a pulse of low concentrations of H_2_O_2_ [94]. Moreover, the mechanisms of ROS-dependent signaling include p38 mitogen-activated protein kinase (MAPK) activation and nuclear translocation of the cardiac transcription factor myocyte enhancer factor 2C (MEF2C) [94]. Also, phosphatidylinositol 3-kinase (PI-3-kinase) appears to play a role in the regulation of the intracellular redox state and to be involved in cardiomyocyte differentiation of embryonic stem cells, since the PI-3-kinase inhibitors LY294002 and wortmannin downregulated ROS and abolished cardiac commitment in embryoid bodies [88]; on the other hand, coadministration of prooxidants with LY294002 was able to resume cardiomyocyte differentiation [88]. PI-3-kinase is also a critical downstream effector of *β*1 integrin signaling, which is activated by mechanical strain-induced ROS and mediates the translocation of *β*-catenin into the nucleus, leading to increased connexin 43 and Nkx 2.5 protein levels required for cardiomyocyte differentiation [95]. ROS generated from NADPH oxidase are also involved in cardiotrophin-1-induced proliferation of cardiomyocytes differentiated from murine embryonic stem cells, acting as signaling molecules in a signaling pathway involving NF*κ*B, Janus kinase signal transducer-2 (Jak-2), signal transducer and activator of transcription-3 (STAT-3), and ERK1/2 [96]. Alterations in antioxidant balance may be also involved in the regulation of cardiomyocyte differentiation. For example, inhibition of redox effector protein-1 (Ref-1), which mediates DNA repair and redox regulation of several transcription factors, followed by treatment with low levels of H_2_O_2_, leads to increases in the intracellular levels of ROS and p53 and induction of cardiac differentiation in adult cardiac stem cells [90].

#### 3.1.2. Excitation-Contraction Coupling

In the heart, excitation-contraction coupling (ECC) is the central mechanism by which electrical activation is translated into cardiac contraction. The events that occur in ECC are well-defined and mainly depend on intracellular Ca^2+^ levels, through the involvement of Ca^2+^ channels and transporters. The depolarization produced by the action potential opens L-type Ca^2+^ channels located on the surface membrane and transverse tubules (T-tubule). The resulting entry of small amounts of Ca^2+^ produces a large increase of [Ca^2+^]_i_ in the dyadic space (the region bounded by the T-tubule and sarcoplasmic reticulum [SR]), which triggers a process termed calcium-induced calcium release via the SR Ca^2+^ release channels (ryanodine receptors [RyR2]), so that a much larger amount of Ca^2+^ from the SR is released. The binding of Ca^2+^ to troponin C establishes actomyosin crossbridge cycling and contraction. During relaxation, Ca^2+^ is removed from the cytoplasm through the involvement of the SR Ca-ATPase (SERCA), other sarcolemmal ion pumps, and exchangers, among which the sodium-calcium exchange (NCX), following ion fluxes between the cytoplasm and mitochondria [40, 65, 97]. Several components of the cardiomyocyte ECC machinery are redox-sensitive targets, and protein modifications include formation of disulfide bounds, thiol nitrosylation and glutathiolation, tyrosine nitration, and phosphorylation [65]; the first modifications refer to channels and ion transporters, the latter modification concerns redox-activated protein kinases [65]. For example, protein kinase A (PKA) exists as a tetramer comprising two catalytic and two regulatory subunits (RI and RII). Under basal conditions, the RI subunits exist as a dimer, but they are not covalently linked; when cellular H_2_O_2_ concentration is elevated, a disulfide bond forms between RI subunits, and this event promotes the translocation and association of PKA with specific A-kinase anchoring proteins (AKAPs), so that PKA is brought close to its substrate [98]. In cardiac tissue, the redox changes induce subcellular translocation and activation of PKA, resulting in phosphorylation of multiple PKA substrates and consequent myocyte contraction. Interestingly, these oxidant-induced modifications occur without elevations in cAMP [98].

The cardiac RyR2 is one of the well-characterized redox-sensitive ion channels in the heart, and modulation of RyR2 activity is mediated by the redox modification of sulfhydryl groups of cysteine residues [99]. It has been shown that O_2_^−^ [98, 100] and H_2_O_2_/OH^−^ [101–103] increase the open probability of cardiac RyR2, this effect being reversed by agents that reduce thiol groups, such as DTT. However, the effects of ROS are likely to depend upon their concentration and duration of exposure. Increased RyR2 oxidation, after acute low-level exposure to ROS, contributes to the enhanced RyR2 activity and SR Ca^2+^ leakage [99]. On the other hand, there is also evidence that after initial stimulation of channel activity, ROS can cause irreversible inactivation of these channels [104] and that excessive oxidation of RyR2 is associated with irreversible activation and increased Ca^2+^ leakage [105]. Abnormal RyR2 function is recognized as an important factor in the pathogenesis of heart failure [105].

Regarding SERCA2a, ROS impairs the oligomerization of PLN altering the PLN-SERCA2a inhibitory interaction, thus enhancing SERCA2a Ca^2+^ transport activity. The ROS-induced changes on PLN are thiol-sensitive and involve the formation of cysteine-based disulfide bonds between PLN monomers, favoring PLN oligomer formation and dissociation from SERCA2a [106]. However, other data suggest that elevated oxidative stress may induce oxidative modifications on SERCA2a, leading to abnormal function of this protein in human diseases, including the metabolic syndrome (MetS). Indeed, myocytes from MetS rats exhibited elevated basal production of ROS accompanied by reduced cytosolic Ca^2+^ removal, which was associated with a significant decrease in SERCA2a-mediated Ca^2+^ reuptake and increased SERCA2a oxidation. Importantly, myocytes from MetS rats treated with the antioxidant N-acetylcysteine (NAC) showed normal ROS levels and SERCA2a-mediated Ca^2+^ reuptake, as well as accelerated cytosolic Ca^2+^ removal, suggesting that elevated oxidative stress may induce oxidative modifications on SERCA2a leading to abnormal function of this protein in this condition [107].

### 3.2. Pathophysiology of Redox Signaling Pathways in Cardiac Diseases

#### 3.2.1. Cardiomyocyte Apoptosis

Cardiomyocyte apoptosis plays an important role in the development of cardiovascular diseases (CVD). Laviola et al. have demonstrated that exposure of human CPC isolated from human heart biopsies to H_2_O_2_ triggers apoptosis by inducing JNK phosphorylation and its nuclear translocation [108]. ROS generation appears to also contribute to palmitate-induced apoptosis in human CPC. Indeed, incubation of human CPCs with palmitate for 16 hours enhanced ROS production, whereas pretreatment with NAC, a precursor compound for glutathione formation, resulted in both reduced palmitate-induced ROS production and apoptosis [109]. Marked expression of CYP2E1, one of the cytochrome P450 isoforms, as well as an effective generator of ROS, is associated with several cellular markers of oxidative stress, including products of lipid peroxidation, and with increased cardiomyocyte apoptosis both *in vitro* and *in vivo* [110]. Generation of ROS following activation of the renin-angiotensin system (RAS) is also involved in the development of CVD. For example, angiotensin II triggers apoptosis in rat H9c2 cells by inducing NADPH oxidase, so that ROS production is increased severalfold, as well as p38 MAPK and expression of caspase-3 [111]. Cardiomyocyte apoptosis also represents an important contributor to hypertrophic remodeling and cell dysfunction [112]. In this context, apoptosis signal-regulating kinase 1 (ASK1) is a redox-sensitive kinase that plays an important role in oxidative stress-induced apoptosis, since ASK1 knockout mice show smaller increases in LV end-diastolic and end-systolic size, smaller decreases in fractional shortening, and lower levels of cellular apoptosis after coronary artery ligation or thoracic transverse aortic constriction compared with wild-type mice [113]. Conversely, overexpression of a constitutively active mutant of ASK1 led to activation of NF*κ*B, a ROS-sensitive transcriptional factor, and cardiac hypertrophy in isolated rat neonatal cardiomyocytes [114]. Another important redox-sensitive kinase is represented by calcium/calmodulin- (Ca^2+^/CaM-) dependent protein kinase II (CaMKII); its activity is involved in ECC and is enhanced not only by Ca^2+^/CaM but also by prooxidant conditions, including elevated angiotensin II. This can induce oxidation of methionine residues (M281/282) in the CaMKII regulatory subunit, leading to cardiomyocyte apoptosis, both *in vitro* and *in vivo* [115], atrial fibrillation [101], and diabetes-related bradycardia after myocardial infarction [102]. CaMKII oxidation is reversed by methionine sulfoxide reductase A (MsrA), and MsrA^−/−^ mice show exaggerated CaMKII oxidation and myocardial apoptosis, impaired cardiac function, and increased mortality after myocardial infarction [115].

#### 3.2.2. Cardiac Hypertrophy

A chronic increase in cardiac workload results in significant expansion of cardiomyocytes, resulting in increased chamber mass and wall thickness, the typical aspects of cardiac hypertrophy. In “physiological” hypertrophy (such as that following exercise training or pregnancy), the myocardial modifications are due to increased workload and are aimed at the maintenance of a normal contractile function to prevent long-term adverse effects. By contrast, “pathological” hypertrophy, as in patients with chronic hypertension or following myocardial infarction, is associated with contractile dysfunction, heart failure, and profibrotic changes in the extracellular matrix [65]. An increasing body of evidence suggests that exogenous ROS may promote cardiac hypertrophy. ERK1/2, JNK, p38 MAPK, and PI-3-kinase/protein kinase B (Akt) represent the main ROS-activated intracellular signaling pathways in cardiomyocytes; these are described in detail elsewhere [116]. Here, we will focus on modulation of hypertrophic signaling pathways by endogenous ROS.

G-protein-coupled receptor (GPCR) agonists, including angiotensin II, endothelin-1, and *α*-adrenoreceptor agonists, can trigger cardiomyocyte hypertrophy via endogenous ROS generation, mainly from Nox2, and subsequent activation of ERK1/2 and NF*κ*B [58, 114, 117–121]. Angiotensin II has important effects on the development and progression of pathological cardiac hypertrophy since it can activate Nox2 by facilitating the complex formation between Nox2 and its cytosolic activators [117]. Indeed, in neonatal cardiomyocytes, a small interfering RNA (siRNA) directed against Nox2 prevents angiotensin II-induced O_2_^−^ generation and cardiomyocyte hypertrophy [120]. Similarly, cardiac hypertrophy after the infusion of angiotensin II for 2 weeks is attenuated in mice with systemic deletion of Nox2 [117]. Akt also plays an important role in angiotensin II-induced cardiomyocyte hypertrophy, since pretreatment of cardiomyocytes with a dominant-negative Akt mutant abrogates angiotensin II-induced cellular hypertrophy [120]. The Nox2/vascular peroxidase 1 (VPO1)/hypochlorous acid (HOCl)/ERK1/2 redox signaling pathway is also implicated in the pathogenesis of angiotensin II-induced cardiac hypertrophy [122]. VPO1 is a peroxidase in the cardiovascular system that uses H_2_O_2_ derived from coexpressed Nox to produce HOCl and catalyze peroxidative reactions.

Pressure overload hypertrophy involves multiple stimuli, including mechanical strain and the activation of G-protein-coupled receptors and other receptors. Angiotensin II also appears to contribute to pressure overload-induced cardiac hypertrophy in rats subjected to abdominal aorta banding by upregulating NADPH oxidase expression and promoting ROS synthesis [123]. Indeed, treatment with apocynin, a natural organic compound structurally related to vanillin and an inhibitor of NADPH oxidase, for 8 weeks reduces the left ventricle/body weight ratio (LV/BW) and atrial natriuretic factor (ANF) mRNA expression, NADPH oxidase activity, and ROS levels [123]. Similarly, allicin, a compound that exhibits antimicrobial, antioxidant, and antiproliferative activity, protects cardiac function and prevents the development of cardiac hypertrophy, both *in vitro* and *in vitro*, by suppressing NADPH oxidase activity and ROS generation, as well as ROS-dependent ERK1/2, JNK1/2, and Akt signaling [124]. The restoration of antioxidant systems represents another strategy to prevent pressure overload-induced cardiac hypertrophy. In male C57BL/6 mice with aortic constriction, short-term caloric restriction is able to attenuate the increase in LV wall thickness, myocyte hypertrophy, and fibrosis, as well as the induction of brain natriuretic peptide (BNP) and collagen III expression, by enhancing myocardial glutathione peroxidase and superoxide dismutase activities [125]. In a similar way, lycopene, a kind of carotenoid antioxidant shown to protect the cardiovascular system, inhibits cardiac hypertrophy, both *in vivo* and *in vitro*, by restoring the impaired antioxidant response element (ARE) activity and activating ARE-driven expression of antioxidant genes [126].

Autophagy represents a self-digesting mechanism responsible for removal of damaged organelles, misfolded proteins during biosynthesis, and nonfunctional long-lived proteins by lysosomes [127], and it is required for the maintenance of cardiomyocyte homeostasis. Autophagy can play positive or negative roles, respectively, in pressure overload-induced cardiac hypertrophy. Zhao et al. have demonstrated that protein kinase D (PKD) can prevent the development of pressure overload-induced cardiac hypertrophy by inhibiting cardiac autophagy via the Akt/mTOR pathway in mice subjected to transverse aortic constriction [128]. On the other hand, it was shown that irisin, a newly discovered myokine, can protect against pressure overload-induced cardiac hypertrophy by inducing autophagy via mTOR-independent activation of the AMPK-ULK1 signaling, both *in vivo* and *in vitro* [129]. However, excessive autophagy plays a maladaptive role in pressure overload-induced hypertrophy. Relevant to this concept, Cao et al. reported that stachydrine, a major constituent of Leonurus heterophyllus Sweet, can inhibit hypertrophy by decreasing angiotensin II-induced excessive autophagy and Nox2 activity in H9c2 cardiomyocytes and can ameliorate transverse aortic constriction-induced cardiac hypertrophy and excessive autophagy in Wistar rats *in vivo* [130].

Endothelin-1 (ET-1) can also promote cardiac hypertrophy. Indeed, stimulation with ET-1 for 4 days induces cell hypertrophy in H9c2 cardiomyocytes, as demonstrated by enhanced expression of the hypertrophic markers BNP and ANF [131]. Conversely, KMUP-1, a synthetic xanthine-based derivative, attenuates ET-1-induced cardiomyocyte hypertrophy through inhibition of the ERK1/2, calcineurin/NFATc4, and RhoA/ROCK pathways and upregulation of heme-oxygenase-1 (HO-1), a stress-response enzyme implicated in cardioprotection [131]. Moreover, *in vivo*, ET-1-induced cardiac hypertrophy leads to heart failure because of the imbalance of multiple parameters, including free radical-induced oxidative stress and antioxidative enzymes such as SOD [132]; indeed, NAC plays a role against ET-1-induced cardiac hypertrophy via SOD regulation [132].

Among GPCR agonists involved in cardiomyocyte hypertrophy, also *α*-adrenoreceptor agonists can promote hypertrophy in adult rat ventricular myocytes via ROS-dependent activation of the Ras-Raf-MEK1/2-ERK1/2 signaling pathway and a thioredoxin-1-sensitive posttranslational oxidative modification of specific cysteine thiols on the small G-protein Ras [133].

#### 3.2.3. Myocardial Ischemia-Reperfusion

The ischemia-reperfusion (I/R) injury is a central mechanism of major cardiovascular diseases, including stroke and myocardial infarction, in which the blood supply to an organ is disrupted and then restored [134, 135]. Increasing evidence suggests that, despite limited O_2_ supply, ROS accumulate rapidly at the beginning of ischemia, generated by the impairment of the mitochondrial respiratory chain [136], activation of xanthine oxidase (XO) [137], and oxidation of ferrous heme (Fe^2+^) in the oxymyoglobin complex [138]. Specifically, during ischemia, Fe^2+^ is converted into Fe^3+^, followed by O_2_^−^ production [138]. Reperfusion is then associated with a burst of ROS production [139–142], mainly from XO and neutrophils [136]. In the presence of XO and O_2_, hypoxanthine can be converted to xanthine and O_2_^−^ [143]; additionally, neutrophils are recruited and activated following I/R, releasing toxic oxidants to the myocardium [144]. Reperfusion also triggers the opening of mitochondrial permeability transition pores (mPTP), which results in cell swelling and rupture, cytochrome c release from mitochondria and initiation of apoptotic cascades, hydrolysis of mitochondrial ATP and lowering of ATP-driven Ca^2+^ pump rates, Ca^2+^ overload, and cell necrosis [145, 146]. Opening of the mPTP causes irreversible damage to the heart during reperfusion after a prolonged period of ischemia [147–150]. This pore is a high-conductance nonspecific channel in the inner mitochondrial membrane that is regulated by multiple pathophysiological effectors [151]; mPTP formation is inhibited by low pH during ischemia, while restoration of pH with mitochondrial calcium overload and excessive ROS generation causes opening of mPTP, occurring soon after reperfusion [151–153]. In addition, a matrix-facing Ca^2+^ binding site is essential to trigger mPTP opening; interestingly, the *Ppif* gene product, cyclophilin D (CyP-D), a propyl isomerase located within the mitochondrial matrix, facilitates mPTP opening during oxidative stress by binding to the inner mitochondrial membrane and augmenting its calcium sensitivity [154]. Moreover, Cyp-D is the mitochondrial receptor for cyclosporine A (CsA), which has a cardioprotective role [151]. Studies carried out by comparing hearts from p66^shc^ knockout and wild-type mice have highlighted the cardioprotective effects elicited by p66^shc^ ablation, since this protects against I/R insults, as demonstrated by reduced release of lactate dehydrogenase in the coronary effluent and by decreased oxidative stress with reduction of malondialdehyde formation and tropomyosin oxidation [155]. Also, accumulation of the citric acid cycle intermediate succinate is responsible for mitochondrial ROS production during reperfusion [156]. Specifically, ischaemic succinate accumulation arises from reversal of succinate dehydrogenase, which in turn is driven by fumarate overflow from purine nucleotide breakdown and partial reversal of the malate/aspartate shuttle. After reperfusion, the accumulated succinate is rapidly reoxidized by succinate dehydrogenase, driving extensive ROS generation by reverse electron transport at mitochondrial complex I [156]. Hence, rapid complex I reactivation has been identified as a central pathological feature of ischemia-reperfusion, and it has been demonstrated that S-nitrosylation of a cysteine of complex I slows the reactivation of mitochondria during the crucial first minutes of the reperfusion [157].

Despite the significant adverse effects of ROS overproduction, a large body of data suggests that low levels of ROS are essential for cardioprotection [158]. For example, it has been demonstrated that both Nox2 and Nox4 are upregulated in response to I/R, thereby contributing to ROS production and consequent myocardial injury. However, not only excessive activation but also suppression of Nox2 and Nox4 below physiological levels is able to exacerbate myocardial I/R injury, whereas a minimum level of ROS production by either Nox2 or Nox4 is essential for the activation of hypoxia-inducible factor-1*α* (HIF-1*α*) and inhibition of PPAR*α* during I/R [158]. HIF-1*α* is a master regulator of hypoxia-regulated gene expression regulated by prolyl hydroxylases (PHDs); under normal oxygen levels, PHDs hydroxylate HIF-1*α*, allowing von Hippel Lindau (VHL) to ubiquitinate HIF-1*α* for its proteasomal degradation. Conversely, in the presence of oxidative stress, PHDs are inactivated, and HIF-1*α* translocate to the nucleus, where it binds to hypoxia response elements (HREs) on the DNA in order to upregulate multiple genes, including glycolytic genes (pyruvate dehydrogenase kinase-1 (PDK1) and hexokinase II (HKII)), important for ATP supply during ischemia, and genes involved in angiogenesis and red blood cell production [145]. Moreover, it has been demonstrated that HIF-1*α* stabilization using either a pharmacological (i.e., chemical inhibition of PHD) or a genetic approach (i.e., cardiac-specific ablation of VHL) protects the heart against I/R injury by promoting glycolysis, decreasing mitochondrial oxidative stress, activating HKII, and inhibiting mPTP opening and DNA damage [159, 160]. Similarly, constitutive overexpression of HIF-1*α* in the murine myocardium results in reduced infarct size; increased capillary density, vascular endothelial growth factor (VEGF), and iNOS expression in peri-infarct and infarct regions; and improved cardiac function [161].

#### 3.2.4. Ischemic Preconditioning

Ischemic preconditioning (IPC) is the most protective intervention against myocardial I/R injury to date [136, 162]. The concept of IPC was introduced in 1986 by Murry et al., who first described the cardioprotective effect of multiple brief ischemic episodes before a subsequent sustained ischemic insult in experimental models with myocardial infarction [163]. Specifically, this protocol revealed that infarct size was reduced by 75% in dogs exposed to four cycles of 5-minute coronary artery occlusions followed by 5 minutes of reperfusion before the onset of 40 minutes of coronary occlusion and 4 days of reperfusion. The mechanisms relative to these endogenous cardioprotective phenomena include triggers, mediators, and effectors [164]. During the initial preconditioning period, ROS or redox signaling activate cardioprotective signal transduction pathways through the involvement of surface receptors, such as A2b adenosine receptor (A2bAR), bradykinin, and opioid-signaling kinases (i.e., PI 3-kinase, Akt, eNOS, JAK, STAT3, and PKC) [165], and posttranslational modification of redox-sensitive proteins [166–172]. The effects of the first phase of IPC last 1-2 hours, after which the protection wanes. Conversely, the late phase of IPC is characterized by upregulation of genes with a protective role, including HIF-1*α*, which plays a central role through the generation of low amounts of mitochondrial ROS acting as signaling messengers [173]. The opening of mitochondrial ATP-sensitive K^+^ (mitoK_ATP_) channels, a protein complex that accounts for ATP-sensitive K^+^ transport across the inner mitochondrial membrane [174], represents another mechanism for this protection in cardiac muscle [175]: the activation of mitoK_ATP_ triggers mitochondrial ROS formation, which in turn inhibits mPTP opening via PKC activation, and prevents cell death [176]. Activation of mitoK_ATP_ also prevents mitochondrial matrix contraction, which could induce beneficial effects by improving ATP synthesis [141]. Experimental evidence demonstrated that also the nuclear factor erythroid 2-related factor 2 (Nrf2)/ARE pathway contributes to the protective effect of the late phase of IPC through upregulation of antioxidant enzymes (i.e., HO-1 and SOD2) [151, 177]. The late preconditioning occurs 24 hours following the initial preconditioning ischemia and lasts for 48-72 hours [178]. The cellular and paracrine effects of preconditioning in cardiomyocytes include the induction of angiogenesis and progenitors, stem cell activation, and alleviation of inflammation and adverse remodeling [179]. Experimental evidence also suggests that the two phases of IPC differ in the cardioprotective actions, since early IPC protects against myocardial infarction while late IPC preserves myocardial cell survival and postischemic LV function [178]. Therefore, the second window of IPC could have a greater clinical implication.

Multiple pharmacological agents are able to induce myocardial IPC, including NO-related agents [180], phosphodiesterase inhibitors [181], adenosine monophosphate-activated protein kinase activators [182], adenosine [183, 184], bradykinin [185], opioid agonists [186, 187] and other G-protein agonists [188, 189], muscarinic agents [190, 191], angiotensin AT_1_ agonists [192], and endothelin [193, 194]. In addition, a number of noxious stimuli (heat stress, rapid pacing, ROS, cytokines, and endotoxins) can trigger myocardial preconditioning [195]. On the basis of this large evidence, future studies will have to consider the use of gene therapy to induce a long-lasting and permanent preconditioning phenotype in the heart.

Recently, it has been discovered that also brief, intermittent ischemia of distant organs, such as the skeletal muscle [196] and kidney [197], can elicit the same protective effects of IPC of the local coronary artery of interest. This phenomenon is defined as “remote IPC” (RIPC). In terms of mechanisms, RIPC depends upon the same signaling pathways and second messengers of traditional IPC. Moreover, there are three hypotheses regarding the central mechanism responsible for RIPC [195]. The neural hypothesis proposes that preconditioning of the organ or tissue remotely from the heart generates endogenous substances, such as adenosine, bradykinin, or calcitonin gene-related peptide (CGRP), which then activate a local afferent neural pathway stimulating an efferent neural pathway, which terminates at the heart and mediates cardioprotection [198]. The humoral hypothesis proposes that the endogenous substances released from the remote organ or tissue enter the bloodstream and activate their respective receptors in the myocardium, thereby triggering the multiple intracellular pathways of cardioprotection implicated in traditional IPC [198]. The third hypothesis proposes that transient ischemia and reperfusion of an organ or tissue provokes a systemic protective response, which suppresses inflammation and apoptosis [198]. Recent data suggest that also the activation of MAPKs (p38 MAPK, ERK1/2, and JNK) within the remote organ might contribute to RIPC-induced cardioprotection [198]. It is not known which of these hypotheses may account for the RIPC-mediated cardioprotective effects and if they interact with each other.

#### 3.2.5. Heart Failure

Heart failure (HF) can be defined as a condition of inadequate cardiac function to maintain systemic perfusion rates appropriate to the body requests under physiological conditions or during increased demand and is often associated with arrhythmias, hypertrophy, and cardiomyocyte death. Despite advances in understanding of the pathophysiology and treatment, HF continues to be a major cause of morbidity and mortality worldwide. Aging, genetic predisposition, traditional risk factors (smoking, diabetes, high cholesterol, and hypertension), and environmental risk factors (air pollution and noise) can induce oxidative stress in vessels [199]. As described above, low ROS levels can produce protective effects, by triggering physiological redox signaling, but when they exceed the cellular antioxidative capacity, cell damage, endothelial dysfunction, and atherosclerosis ensue [16, 200–202]. These biological events, in association with ischemia and myocardial infarction, can result in loss of functional myocardium and subsequent decrease of cardiac output [199]. Additional oxidative stress in the heart, triggered by neuroendocrine activation mediated by the RAS and sympathetic nervous system, may contribute to the onset and progression of heart failure [199, 203, 204], involving increased preload and afterload, receptor-induced activation of Nox2, and mitochondrial dysfunction [199, 205]. Specifically, mitochondria amplify Nox-induced ROS and may act as “redox hubs” [206, 207]. Furthermore, multiple studies have also shown that mitochondrial ROS affect a broad range of cellular functions in the context of heart failure. Indeed, excessive ROS trigger mPTP opening and cause cell death, mitochondrial DNA damage, and impaired mitochondrial biogenesis [23, 208, 209]. In turn, mitochondrial ROS act as molecular mediators of hypoxia signaling, MAP kinase pathway, and inflammation [210, 211]. The specific role of these mechanisms during the development of heart failure needs to be fully elucidated. On the other hand, a reduction in SOD, catalase, and glutathione peroxidase may represent contributing factors [212, 213]. Manganese SOD knockout mice exhibit extensive mitochondrial injury within degenerating cardiomyocytes and markedly enlarged and dilated hearts, suggesting that deficiency of this antioxidant causes increased susceptibility to oxidative mitochondrial injury in cardiac myocytes after postnatal exposure to ambient oxygen concentrations [214]. Likewise, overexpression of glutathione peroxidase in an animal model of myocardial infarction attenuates LV cavity dilatation and dysfunction, increases LV end-diastolic pressure, and improves LV function, by decreasing myocyte hypertrophy, apoptosis, and interstitial fibrosis after myocardial infarction [215].

It may be puzzling that large clinical trials failed to show beneficial effects of antioxidants such as vitamins E and C [205, 216]. This can be explained by the absence of specificity of vitamins for sites where ROS generation occurs in heart failure (i.e., mitochondria) and/or the ability of vitamins to interact with ROS much more slowly than ROS with their direct counterpart NO [216]. Moreover, both administration of high doses of vitamin E and long-term vitamin E treatment are deleterious for vascular [217, 218] and myocardial [219, 220] functions. Specifically, the GISSI-Prevenzione trial explored the effect of vitamin E on development of heart failure in 8415 postinfarction patients without heart failure at baseline and followed up for 3.5 years; it showed that vitamin E treatment was associated with a significant 50% increase of heart failure in patients with LV dysfunction (ejection fraction < 50%) [221]. Another controlled clinical trial, conducted in 56 patients with advanced heart failure (New York Heart Association functional class III or IV) for 12 weeks, demonstrated that vitamin E supplementation did not result in any significant improvement of prognostic or functional indexes of heart failure or in the quality of life of these patients [222]. Moreover, the HOPE-TOO trial, a large randomized double-blind, placebo-controlled trial conducted in patients with vascular disease or diabetes mellitus, showed that long-term vitamin E supplementation does not prevent major cardiovascular events and may even increase the risk and hospitalization for heart failure [220]. Potential explanations could be linked to the ability of multiple antioxidants to act synergistically, so vitamin E would be effective only if used in combination with other micronutrients [223]. Other authors have suggested that vitamin E has prooxidant effects in the absence of coantioxidants *in vivo* [224]. Moreover, vitamin E has no effect on specific ROS species, such as hypochlorite-induced oxidative species, and may interfere with lipoprotein metabolism diminishing high-density lipoprotein-2 cardioprotective effects [225].

In the last years, the importance of ROS compartmentalization has also been recognized; thus, specific treatments able to target the most relevant compartment for ROS production appear to be more promising. There are three strategies for mitochondrial pharmacology. The first is by targeting compounds to mitochondria, by conjugation to a lipophilic cation, such as triphenylphosphonium (TPP), allowing the selective uptake of the attached bioactive moiety into the mitochondrial matrix [226]. The most prominent example of this strategy is MitoQ, where a ubiquinone derivative is coupled to TPP [227]. Specifically, MitoQ improves mitochondrial dysfunction in heart failure induced by pressure overload by decreasing hydrogen peroxide formation, improving mitochondrial respiration, and mPTP opening [228]. Other peptides that could be used include the Szeto–Schiller (SS) peptides [229] and the mitochondrial-penetrating peptides (MPPs) [230]. Both classes of peptides comprise a mix of cationic and hydrophobic alkyl or aromatic amino acid residues that are taken up by mitochondria [229, 230]. The second strategy consists in the use of untargeted mitochondrial drugs, compounds which are not targeted to mitochondria but act there through the binding to specific mitochondrial targets [226]. These drugs include cyclosporine A (CsA), which inhibits mPTP; dichloroacetate (DCA), which activates pyruvate dehydrogenase complex; and AICAR, which acts as AMPK agonist increasing mitochondrial biogenesis. Finally, a third way to target mitochondrial ROS is to interfere with cellular ion handling, for example, by using CGP37157, which inhibits the mitochondrial Ca^2+^/Na^+^ exchanger preventing oxidation of NADH and NADPH, emission of ROS, maladaptive cardiac remodeling, and arrhythmias in animal models of heart failure with reduced ejection fraction [231, 232].

It has also been demonstrated that hydralazine/nitrate (i.e., nitroglycerin or isosorbide dinitrate (ISDN)) combination therapy has beneficial effects on morbidity and mortality in patients with heart failure, improving the balance between O_2_^−^ and NO, which is impaired in this condition [233]. Moreover, hydralazine also reduces the Ca^2+^ leakage from the SR and improves Ca^2+^ cycling and contractility in failing cardiac myocytes [234].

Finally, other heart failure therapies, such as angiotensin-converting enzyme (ACE) inhibitors, have antioxidant effects, since they are able to ameliorate inflammatory processes in the vessel wall [235] and to prevent smooth muscle cell proliferation and activation of NADPH oxidase [235, 236]; they are usually used instead of hydralazine avoiding a thrice-a-day drug with many adverse side effects [237].

#### 3.2.6. Chemotherapy-Induced Cardiotoxicity

Chemotherapy-induced cardiotoxicity is a serious complication that limits the clinical use of chemotherapeutic agents, particularly the anthracyclines. Anthracyclines include doxorubicin (DOX), daunorubicin, epirubicin, and idarubicin, which are highly effective against acute lymphoblastic and myeloblastic leukemias. Anthracycline-induced cardiotoxicity can occur in an acute or chronic manner. The acute cardiotoxicity is reversible and occurs either during treatment or immediately afterwards and leads to acute myocarditis and arrhythmia, while the chronic cardiotoxicity can occur even many years after the end of treatment, affects mortality, and requires long-term therapy [238]. DOX-derived ROS impair multiple cellular processes, including cellular hypertrophy, extracellular matrix remodeling, alterations of cardiac contraction, and cardiac cell death, leading to cardiomyopathy and heart failure. DOX-induced cardiac hypertrophy is significantly inhibited in the cardiac-specific metallothionein- (MT-) overexpressing transgenic mice, suggesting that oxidative stress is critically involved in the DOX-induced hypertrophy [239]. Extracellular matrix microenvironment plays an important role in the myocardium, since it provides a platform for cardiomyocytes to attach, align, and orient and for cellular contraction. Impairment in its structure may affect heart function [240–242]. DOX increases metalloproteinase- (MMP-) 2 and MMP-9, two enzymes that are responsible for the extracellular matrix degradation and that are activated by ROS, through the involvement of p38 MAPK and NADPH oxidase [243]. Moreover, Nox2^−/−^ mice exhibited a less pronounced cardiac remodeling associated with DOX chemotherapy when compared to the wild-type controls, further suggesting that ROS specifically derived from the Nox2 NADPH oxidase give a relevant contribution to the development of cardiac remodeling associated with chemotherapy-induced cardiotoxicity [244]. DOX-induced ROS also downregulate the activity of GATA4, a transcription factor critical for regulation of cardiac differentiation, leading to myofibrillar deterioration, reduction of contractile function [245, 246], and impaired calcium homeostasis. The latter effect is due to DOX-derived, ROS-induced lipid peroxidation of membrane lipids and, consequently, to impairment of the function of membrane-bound proteins, including mitochondrial calcium channel [238]. Additionally, Arai et al. found that DOX induces a decrease in SERCA2 mRNA levels in cultured rat neonatal cardiac myocytes, through the involvement of the transcription factor Egr-1 and p44/42 MAPK, leading to impaired calcium handling [247]. DOX-induced cytosolic Ca^2+^ overload also results in calcineurin activation, increased transcription of Fas ligand (FasL), and activation of extrinsic pathway [248]. DOX-induced ROS are also able to inhibit the expression of the caspase-8 inhibitory protein FLIP, thus triggering apoptosis [249]. Furthermore, DOX-induced oxidative stress activates p53, MAPKs, and NF*κ*B, causing an alteration in the ratio of proapoptotic to antiapoptotic proteins (i.e., an increase in Bax to Bcl-2 ratio) [250, 251]. DOX is able to trigger apoptosis also by inducing directly cytochrome c release and caspase-3 activation [252, 253].

Multiple new clinical trials on the benefit-risk of dexrazoxane have been published. These trials, in conjunction with older trials, indicate that dexrazoxane is well-tolerated and can prevent anthracycline-associated cardiotoxicity; however, adverse hematological effects remain the most common problem in patients receiving dexrazoxane therapy, which requires routine peripheral blood monitoring in patients [254]. Carvedilol, a beta-adrenergic receptor antagonist with antioxidant properties, has a positive impact on cardiac mitochondria in *in vitro*, *ex vivo*, and *in vivo* models of cardiac dysfunction [255]. Importantly, carvedilol also acts as an inhibitor of mitochondrial complex-I, which has been proposed to be involved in the mechanisms of DOX-induced cardiotoxicity [255]. Indeed, prophylactic use of carvedilol in patients receiving anthracycline allows protection of both systolic and diastolic LV functions [256]. Another clinical study, the OVERCOME trial (preventiOn of left Ventricular dysfunction with Enalapril and caRvedilol in patients submitted to intensive ChemOtherapy for the treatment of Malignant hEmopathies), supports the protective role of carvedilol and, more specifically, the ability of combined treatment with enalapril and carvedilol in the prevention of chemotherapy-induced LV systolic dysfunction in patients with hematological malignancies [257].

#### 3.2.7. Atrial Fibrillation

Atrial fibrillation is a complex and heterogeneous arrhythmia, promoted by electrophysiological and structural abnormalities that characterize atrial remodeling [258]. Multiple risk factors, including hypertension, congestive heart failure, diabetes, coronary heart disease, obesity, and tissue damage (cardiac surgery, acute ischemia, and myocarditis) may lead to the onset of atrial fibrillation, which in turn can lead to blood clots, stroke, heart failure, and other complications [258]. Both experimental and clinical data indicate that oxidative stress is implicated in the pathophysiology of atrial remodeling [259]. Mihm et al. have demonstrated that oxidative modification of myofibrillar proteins is increased in atrial myocytes from atrial fibrillation patients, leading to the loss of fibrillar protein function; this suggests that oxidative stress plays an important role in this setting [259]. *In vivo* data also suggest that peroxynitrite and/or protein nitration may be important mediators of atrial fibrillation; indeed, in patients undergoing cardiac bypass graft surgery, treatment with ascorbate, an antioxidant and peroxynitrite decomposition catalyst, significantly decreases incidence of postoperative atrial fibrillation (POAF) [260]. POAF is a common complication after cardiac surgery that occurs in up to 60% of patients, often as a consequence of cardiopulmonary bypass and cardioplegic arrest [261]. Patients with POAF have increased risk of cardiovascular mortality, stroke, and other arrhythmias [262–265]. It has been proposed that myocardial MAO activity is associated with an increased risk for POAF [266] and that NADPH oxidase and, to a lesser extent, dysfunctional NOS may contribute significantly to superoxide production in the fibrillating human atrial myocardium, as well as to atrial oxidative injury and electrophysiological remodeling [267]. Furthermore, in a prospective study, Montaigne et al. demonstrated that both decreased preoperative mitochondrial respiration and increased sensitivity to calcium-induced mPTP opening are significantly associated with POAF and that the mitochondria/oxidative phosphorylation gene cluster expression is downregulated in preoperative atrial tissue of patients in whom POAF develops [268]. Thus, these data identify the mitochondria as a new potential target for POAF prevention strategies [268].

In multiple clinical studies, drugs with antioxidant properties have been used to reduce the incidence of atrial fibrillation. In a small clinical trial, oral vitamin C administration had a positive effect on early recurrence rates after successful electrical cardioversion of persistent atrial fibrillation and on associated inflammation [269]. In patients scheduled for cardiac surgery with extracorporeal circulation, antioxidant supplementation with n-3 PUFAs and vitamins C and E increased antioxidant potential, attenuated oxidative stress and inflammation, and favorably affected POAF, especially in older patients [270, 271]. The addition of the antioxidant NAC to cardioplegia has been shown to decrease both oxidative stress and POAF incidence in patients undergoing CABG surgery [272, 273]. The same research group has also demonstrated that there is a synergistic effect of NAC plus carvedilol in reducing oxidative stress and POAF incidence when compared with metoprolol or carvedilol alone [273, 274]. Allopurinol, a classic xanthine oxidase inhibitor, has been also shown to suppress atrial fibrillation promotion by preventing both electrical and structural remodeling in a canine model of atrial pacing-induced LV dysfunction [275], and its use for >6 months was associated with a reduced risk of incident AF in the elderly [276]. Accumulating evidence indicates that the Mediterranean dietary pattern allows protection against oxidative stress [277]. Indeed, patients with atrial fibrillation have lower adherence to Mediterranean diet and lower antioxidant intake compared to control population; on the other hand, patients with arrhythmia showing adherence to Mediterranean diet have more probability of a spontaneous conversion of atrial fibrillation [278]. Additionally, the PREDIMED (Prevención con Dieta Mediterránea) trial has suggested that extravirgin olive oil in the context of a Mediterranean dietary pattern may significantly reduce the risk of atrial fibrillation, while no effect was found for the Mediterranean diet with mixed nuts [279]. Moreover, long-term consumption of antioxidant-rich foods, including wine, coffee, and fruits, is associated with a reduced incidence of POAF in patients undergoing cardiac surgery [280].

#### 3.2.8. Diabetic Cardiomyopathy

Diabetic cardiomyopathy (DCM) is a disorder of the heart muscle in patients with diabetes in the absence of other comorbidities related to diabetes, such as hypertension or coronary artery disease [281]. The clinical features of DCM include structural changes of the left ventricle, such as ventricular hypertrophy, fibrosis, reduced ventricular compliance, and diastolic dysfunction [282], and may lead to the typical symptoms of heart failure, including chest pain, elevated blood pressure, shortness of breath on exertion, and ankle edema [283, 284]. Hyperglycemia-, hyperlipidemia-, and inflammation-induced oxidative stress is widely considered one of the major causes of the pathogenesis of this disease. Proteins and lipids are among the first targets for oxidative stress, and in the plasma of type 2 diabetes mellitus (T2DM) patients, the oxidative products of protein and lipid peroxidation and nitric oxide levels are significantly increased [285]. Moreover, the levels of enzymatic (glutathione peroxidase, SOD, and catalase) and nonenzymatic (beta-carotene, retinol, vitamin C and E, and uric acid) antioxidants of erythrocytes show a significant decrease in T2DM patients compared to normal subjects [285].

The main sources of free radicals in diabetic myocardium include mitochondria, NADPH oxidase, and NOS [286]. Experimental evidence sustains the link between multiple disturbances in mitochondrial function and T2DM, including mutations in mitochondrial DNA (mtDNA) and reduction in mtDNA copy number [287]. In veins and arteries of diabetic patients, both the expression and activity of NADPH oxidase protein subunits (p22^phox^, p67^phox^, and p47^phox^) are significantly increased [288]. Rac1, the cytosolic component of many NADPH oxidase isoforms, is also involved in the pathogenesis of diabetic cardiomyopathy; indeed, the effects of hyperglycemia on mitochondrial ROS production in the heart and myocardial dysfunction are significantly decreased in Rac1-knockout mice treated with streptozotocin [289]. In addition, in cultured cardiomyocytes, high glucose upregulates Rac1 and NADPH oxidase activity and induces apoptotic cell death, which are both blocked by overexpression of a dominant negative mutant of Rac1, knockdown of gp91*^phox^* or p47*^phox^*, or NADPH oxidase inhibitor [289]. In *db/db* mice, administration of the Rac1 inhibitor NSC23766 significantly inhibits NADPH oxidase activity and apoptosis and slightly improves myocardial function [289]. Collectively, these data demonstrate that Rac1 plays a central role in NADPH oxidase-dependent ROS production that contributes to myocardial dysfunction in diabetes.

Physiological coupling of eNOS requires its interaction with the cofactor tetrahydrobiopterin (BH_4_), and its reduced availability has been identified in vessels and endothelial cells of experimental animals [286]. Interestingly, degradation of cardiac GTP-cyclohydrolase-I (GTPCH), the enzyme responsible for the synthesis of BH_4_, contributes to the pathogenesis of DCM, since either cardiomyocyte-specific overexpression of GTPCH or inhibition of the 26S proteasome, which is responsible for the degradation of GTPCH proteins, with MG132 protects the heart against DCM by elevating cardiac GTPCH proteins [290]. The beneficial effects of GTPCH on diabetic hearts are associated with an improvement in intracellular Ca^2+^ signaling [290]; these data suggest that cardiomyocyte GTPCH may represent a potent therapeutic target for DCM and that developing novel 26S proteasome inhibitors with specificity towards cardiac GTPCH may be useful for the clinic treatment of DCM. Similarly, oral administration of sepiapterin, a precursor of BH_4_, significantly increases BH_4_ and the BH_4_/BH_2_ ratio and inhibits the formation of malondialdehyde, 4-hydroxy-nonenal, and nitrotyrosine, which are markers of oxidative/nitrosative stress in diabetic heart of eNOS, iNOS, and nNOS knockout mice [291]. However, the increase in NO following sepiapterin treatment, as well as the increase in percentage fractional shortening, is significantly attenuated in the iNOS^−/−^ diabetic mouse heart, suggesting that sepiapterin inhibits uncoupling of NOS and improves left ventricular function by increasing iNOS-derived NO in the diabetic heart [291]. These results are in line with the work of Okazaki et al., which demonstrate that reversal of iNOS uncoupling by BH_4_ treatment increases myocardial tolerance to I/R injury in the diabetic rat heart, through the increase of iNOS-derived NO and the elimination of oxidative stress [292]. Chronic hyperglycemia also leads to glycation, a process in which carbohydrates covalently and nonenzymatically bind to proteins and lipids. Glycation products can combine to form cross-linked structures, known as advanced glycation end products (AGEs), which in turn can bind to cell surface receptors (RAGE), triggering a cascade of events that lead to ROS generation, activation of NF*κ*B, and production of proinflammatory cytokines, thus contributing to diabetic complications [293]. Indeed, serum AGE levels are significantly higher in diabetic patients with vascular complications as compared to diabetic patients without complications [294]. Additionally, RAGE mRNA expression levels are increased in diabetes, yet severalfold higher in diabetic subjects with as compared to those without vascular complications [294]. Chronic treatment with mangiferin, an antidiabetic and anti-inflammatory agent, significantly ameliorates DCM by preventing the release of inflammatory cytokines, ROS accumulation, NF*κ*B nuclear translocation, AGE production, and mRNA and protein expression of RAGE [295]. Another mechanism that contributes to DCM is represented by calpain-1 accumulation in mitochondria, a calcium-activated intracellular proteinase [296]. The increased mitochondrial calpain-1 is associated with mitochondrial ROS generation, oxidative damage, and ATP synthase disruption [296]. Hyperglycemia and proinflammatory stimuli result in enhanced mitochondrial MAO-dependent H_2_O_2_ formation that, in turn, induces mitochondrial dysfunction and endoplasmic reticulum stress [297]. Moreover, MAO-dependent oxidative stress also contributes to mast cell degranulation and cardiac fibrosis, ultimately resulting in diastolic dysfunction in type 1 diabetes [297]. Administration of the MAO inhibitor pargyline prevents exacerbated ROS formation, restores mitochondrial and endoplasmic reticulum homeostasis, and abolishes mast cell degranulation and fibrosis, thus improving LV diastolic function [297]. Lipoxygenases (LOXs) are a ubiquitous family of nonheme iron enzymes involved in the peroxidation of arachidonic acid and linoleic acid, which in the presence of molecular oxygen are converted into a variety of hydroperoxides. Specifically, 12-LOX and 15-LOX convert arachidonic acid into 12- and 15-hydroxyeicosatetraenoic acids, releasing ROS in this process [293]. These enzymes may be activated by hyperglycemia, resulting in increased cardiac oxidative stress and DCM. Indeed, Suzuki et al. have shown that expression of 12/15-LOX and the inflammatory cytokines TNF*α* and NF*κ*B are upregulated in streptozotocin-induced diabetic hearts. In addition, deletion of 12/15-LOX significantly improves diabetic-induced cardiac dysfunction and fibrosis, in parallel with the reduction in TNF*α*, NF*κ*B, and ROS levels in the heart [298].

Lipotoxicity has also recently emerged as an important contributor to the development of cardiac dysfunction associated with obesity and T2DM [299]. Saturated fatty acids (SFA) are known to impair metabolic pathways [299] and to increase apoptosis both in cardiomyocytes [300] and cardiac stem/progenitor cells [109, 301]. Chronic treatment of human cardiac progenitor cells with palmitate, the major SFA in the plasma, triggers ROS generation, which in turn contributes to palmitate-induced apoptosis in this specific class of human cardiac stem cells; indeed, pretreatment with NAC results not only in reduced ROS production but also in reduced palmitate-induced caspase-3 cleavage and cytoplasmic oligonucleosome levels [109]. SFA lead to ROS generation through multiple mechanisms, including PKC-dependent activation of NADPH oxidase [302], mitochondrial uncoupling and beta-oxidation [303], and impairment of the endogenous antioxidant defenses, such as decreased intracellular glutathione [304].

Drugs used in the management of T2DM can improve oxidative stress and antioxidant status in cell cultures, experimental animal models, and trials on patients [305]. The biguanide metformin is one of the most used oral hypoglycemic medications [306]. Metformin therapy has been shown to restore the antioxidant status (intracellular ROS generation and AGE) and inflammatory parameters in T2DM patients [307], and its ability in reducing oxidative stress is more effective compared with lifestyle modification alone [308]. Rosiglitazone, an antidiabetic drug belonging to the thiazolidinedione class, is also able to significantly decrease plasma peroxides in overweight nondiabetic people [309]. Sitagliptin, a dipeptidyl peptidase-4 (DPP-4) inhibitor, the enzyme responsible for inactivating the incretin hormone glucagon-like peptide-1 (GLP-1), exerts protective effects against DCM in streptozotocin-induced diabetic rats [310]. Specifically, sitagliptin has a strong modulatory effect against hyperglycemia-induced cardiac inflammation and oxidative stress, via enhancement of antioxidant defenses and decrease in cardiac lipid peroxidation [310] and also through normalizing malondialdehyde (MDA), advanced protein oxidation product (APOP), and NO concentrations in heart and kidney tissues in two kidney and one clip (2K1C) rats [311].

## 4. Clinical Studies and Trials with Antioxidant Therapies in Cardiovascular Diseases Related with Oxidative Stress

**Figure 1:**
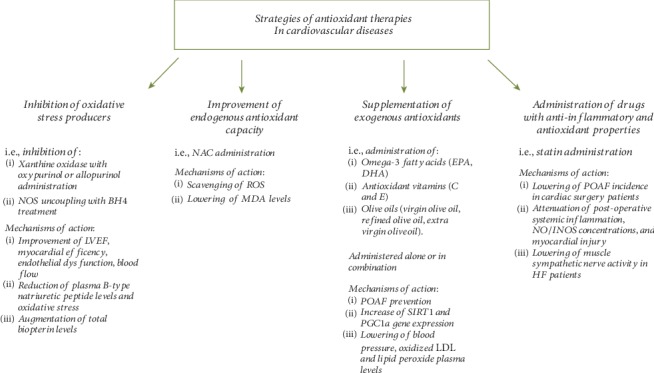
Summary of the main antioxidant therapy approaches in the clinical setting. Multiple studies have assessed different approaches: inhibition of oxidative stress producers (i.e., inhibition of xanthine oxidase with oxypurinol or allopurinol administration and inhibition of NOS uncoupling with BH_4_), improvement of endogenous antioxidant capacity (i.e., NAC administration), supplementation of exogenous antioxidants (i.e., administration of omega-3 fatty acids EPA and DHA, antioxidant vitamins C and E, and olive oils), and administration of drugs with anti-inflammatory and antioxidant properties (i.e., statins). The main mechanisms of action are also described. BH_4_: tetrahydrobiopterin; DHA: docosahexaenoic acid; EPA: eicosapentaenoic acid; HF: heart failure; iNOS: inducible nitric oxide synthase; LVEF: left ventricular ejection fraction; LDL: low-density lipoproteins; MDA: malondialdehyde; NAC: N-acetyl cysteine; NO: nitric oxide; NOS: nitric oxide synthase; PGC1*α*: peroxisome proliferator-activated receptor gamma coactivator gene-alpha; POAF: postoperative atrial fibrillation; ROS: reactive oxygen species; SIRT1: sirtuin-1.

Multiple studies have assessed the potential of antioxidant stress therapies in the clinical setting, by using four different approaches: inhibition of oxidative stress producers (i.e., xanthine oxidase and NOS uncoupling), improvement of endogenous antioxidant capacity (i.e., NAC), improvement of antioxidant capacity by supplementation of exogenous antioxidants (i.e., vitamins A, C, and E), and administration of drugs with anti-inflammatory and antioxidant properties (i.e., statins) (Figure 1).

Administration of the xanthine oxidase inhibitors oxypurinol or allopurinol in patients with chronic heart failure, idiopathic dilated cardiomyopathy, or acute myocardial infarction has been shown to improve LV ejection fraction, myocardial efficiency, endothelial dysfunction, peripheral vasodilator capacity, and blood flow and to reduce plasma B-type natriuretic peptide levels and oxidative stress [37, 312–316]. However, a randomized controlled clinical trial conducted in a larger number of patients (*n* = 405) with NYHA III to IV heart failure has shown that oxypurinol does not improve a clinical composite score comprising heart failure morbidity, mortality, and quality of life [317]. Similarly, inhibition of NOS uncoupling through oral BH_4_ treatment causes an increase of total biopterin levels in patients with established coronary artery disease but has no effect on vascular redox state or endothelial function, owing to systemic and vascular oxidation of BH_4_ [318].

NAC administration has been extensively tested as a strategy to improve endogenous antioxidant capacity. In a randomized, double-blind, placebo-controlled clinical trial conducted in 40 patients undergoing coronary artery surgery and subjected to cardiopulmonary bypass and cardioplegic arrest, NAC has been shown to attenuate myocardial oxidative stress [319]. Interestingly, Rodrigues et al. have demonstrated that NAC as an additive to blood cardioplegia in patients undergoing on-pump coronary artery bypass graft surgery may reduce oxidative stress and the resulting coronary endothelial activation [320]. NAC infusion provides cardiac protection through scavenging of oxygen free radicals levels also in adult patients undergoing elective abdominal aortic aneurysm repair [321]; specifically, NAC reduces MDA levels and increases total antioxidant capacity, as well as postoperative concentrations of myocardial-specific proteins (cardiac troponin-I and creatine phosphokinase-MB) and proinflammatory cytokines (TNF*α* and IL-1*β*) [321]. However, high-dose oral NAC in patients undergoing open-heart surgery had no effect on the incidence of POAF, postoperative hospital staying, morbidity, and mortality [322].

Clinical studies have also assessed the potential of increasing the antioxidant capacity through the supplementation of exogenous antioxidants. Administration of omega-3 fatty acids (eicosapentaenoic acid (EPA) and docosahexaenoic acid (DHA), in 1 : 2 ratio, respectively) and antioxidant vitamins (vitamins C and E) is able to reduce oxidative stress and to prevent connexin-40 and connexin-43 lateralization in atrial tissue, likely contributing to POAF prevention in patients undergoing cardiac surgery. However, it fails to fully prevent POAF occurrence because these compounds have no effects on the normalization of connexin 40 downregulation and connexin 45 upregulation, which may promote POAF [323]. Moreover, omega-3 and vitamin E coadministration has beneficial effects also in coronary artery disease patients by increasing catalase levels and total antioxidant capacity in serum and by augmenting gene expression of SIRT1 and PGC1*α* in peripheral blood mononuclear cells [324]. SIRT1 is a member of the sirtuin family, and it can regulate oxidative stress via affecting p53 [325, 326], while PGC1*α* is a key regulator of mitochondrial respiration playing an important role in metabolism and energy homeostasis [324]. Another clinical trial has examined the antioxidant and antihypertensive effect of two similar olive oils, virgin olive oil (VOO) and refined olive oil (ROO), which differ in their phenolic compounds (PC) (refined: 14.7 mg/kg versus virgin: 161.0 mg/kg) in 40 men with stable coronary heart disease [327]. The main PC in olive oil are oleuropein and ligstroside aglycones, which by hydrolysis give both hydroxytyrosol and tyrosol [328]. Administration of VOO for 3 weeks led to a decrease of oxidized LDL and lipid peroxide plasma levels *in vivo*, together with higher activities of glutathione peroxidase, compared to ROO consumption. Furthermore, systolic blood pressure decreased after intake of VOO in hypertensive patients. These data supported the hypothesis that VOO consumption could provide beneficial effects on CV risk factors [327]. Also the PREDIMED randomized trial has demonstrated that specific dietary patterns might improve CV risk, since high intakes of polyphenols, through use of nuts and extravirgin olive oil, are able to decrease blood pressure in elderly hypertensive populations and consequently their CV risk by increasing plasma NO [329].

However, a large-scale meta-analysis of 50 randomized controlled trials with 294,478 participants suggests that there is no evidence to support the use of vitamin or antioxidant supplements for the primary or secondary prevention of major cardiovascular events [330]. Furthermore, these supplements are not associated with any reduced risk of such events in the subgroup meta-analyses according to various factors, including type of vitamins and antioxidants, type of cardiovascular outcomes, study design, methodological quality, duration of treatment, funding source, provider of supplements, type of control, number of participants in each trial, and supplements given singly or in combination with other vitamins or antioxidant supplements [330]. Taken together, these findings suggest that most antioxidative stress therapies have failed in providing solid evidence of clinical benefits, even though the exact reasons and mechanisms remain unknown. One reason could be that in the experimental settings, the majority of the studies utilize heart failure models to test the efficacy of antioxidative stress treatments, and at this stage, antioxidative stress therapies are probably not capable of limiting the damage already induced by oxidative stress [331]. Other factors that could potentially contribute to the failure of clinical trials include (i) inadequate knowledge of antioxidant pharmacological actions, (ii) insufficient dose-response studies, (iii) the presence of interfering drugs which could affect the pharmacokinetics of antioxidants, (iv) the lack of optimal biomarkers and clinical end points to evaluate the efficacy of antioxidants in cardiac diseases, (v) the limited sample size, (vi) the lack of reproducible studies in different populations across the world [332], and (vii) the possibility that only specific patient populations benefit from antioxidant stress treatment [331]. Noteworthy, the improvement of endogenous antioxidant capacity, through NAC supplementation, today represents the approach with the best results in terms of patient outcomes [319–321]. For this reason, future oxidative stress therapies should probably focus on improving the endogenous antioxidant capacity, rather than inhibition of oxidative stress producers or supplementation of exogenous antioxidant [331].

Statins (HMG-CoA reductase inhibitors) have also proven to reduce cardiovascular events in patients at risk for adverse outcomes by lipid-lowering effects and anti-inflammatory and antioxidative stress properties [333]. The ARMYDA-3 (Atorvastatin for Reduction of MYocardial Dysrhythmia After cardiac surgery) study shows that treatment with atorvastatin, initiated one week before elective cardiac surgery with cardiopulmonary bypass and continued in the postoperative period, significantly decreases the incidence of POAF [334]. Similarly, a pilot double-blind placebo-controlled study has demonstrated that oral pravastatin before nonemergent coronary artery bypass grafting significantly attenuates postoperative systemic inflammatory response and systemic NO/iNOS concentrations and reduces myocardial injury [335]. Additionally, Deo et al. demonstrated that short-term statin therapy (1 month) reduces muscle sympathetic nerve activity along with cholesterol, total ROS, and superoxide in heart failure patients [336]. Furthermore, a meta-analysis of 54 trials supports the evidence for the beneficial effects of preoperative statin therapy in cardiac surgery patients, with lower mortality rates and favorable outcomes including stroke, atrial fibrillation, and length of stay in the intensive care unit and in the hospital [337].

Recently, also sodium-glucose cotransporter 2 (SGLT2) inhibitors, a novel class of antidiabetic drugs, have been shown to decrease cardiac oxidative stress in animal models, independently from their glucose-lowering effects [338]. The results of the Empagliflozin, Cardiovascular Outcomes, and Mortality in type 2 diabetes (EMPA-REG OUTCOME) trial, the Canagliflozin Cardiovascular Assessment Study (CANVAS), and the Dapagliflozin Effect on Cardiovascular Events–Thrombolysis in Myocardial Infarction 58 (DECLARE–TIMI 58) trial showed significant reductions of cardiovascular events with the use of empagliflozin and canagliflozin, respectively, in patients with T2DM and high cardiovascular risk [339–341]. However, these positive effects could not be attributed solely to their antidiabetic effects. Empagliflozin has been proposed to decrease oxidative stress via promoting Nrf2 translocation to the nucleus and activating Nrf2/ARE signaling in type 2 diabetic mice models [342]. In addition, SGLT2 inhibitors may also affect SGLT1 activation, thus reducing serum glucose, and subsequently oxidative stress, resulting in myocardial or neuronal cell damage [343]. The ongoing EMPA-HEART trial will evaluate whether the chronic treatment with the SGLT-2 inhibitor empagliflozin may modulate myocardial oxidative stress plasma biomarkers, such as myeloperoxidase (MPO), in diabetic patients [344].

## 5. Conclusions

**Figure 2:**
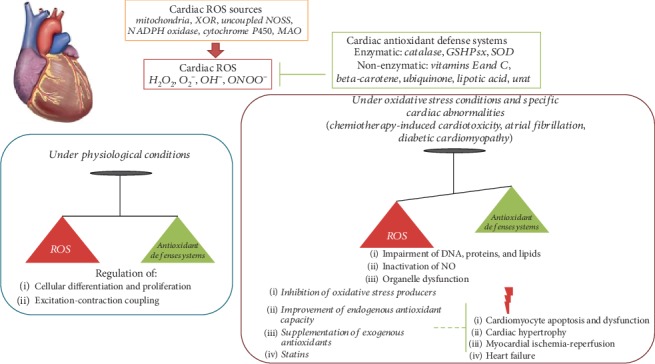
Role of the ROS system in the heart in physiology and disease. ROS are oxygen-based chemical species characterized by high reactivity, and they include H_2_O_2_, OH^−^, O_2_^−^, and ONOO^−^. The most important cardiac ROS sources are mitochondria, xanthine oxidoreductase, uncoupled nitric oxide synthases, NADPH oxidase, cytochrome P450, and monoamine oxidases. Multiple antioxidant defense systems counteract ROS accumulation by scavenging and converting ROS to nontoxic molecules. These systems are both enzymatic and nonenzymatic: enzymes include catalase, glutathione peroxidase (GSHPsx), and superoxide dismutase (SOD) and nonenzymatic antioxidants include vitamins C and E, beta-carotene, ubiquinone, lipoic acid, and urate. ROS represent important second messengers within the heart, since they are involved in multiple physiological processes including differentiation, proliferation, and excitation-contraction coupling. Oxidative stress is defined as a dysregulation between the production of ROS and the endogenous antioxidant defense mechanisms. ROS are also involved in the onset of some complications related to specific clinical settings, including chemotherapy-induced cardiotoxicity, postoperative atrial fibrillation, and diabetic cardiomyopathy. When ROS are in excess, they can induce impairment of DNA, proteins, and lipids; inactivation of NO; and organelle dysfunction leading to cardiomyocyte apoptosis and dysfunction, cardiac hypertrophy, myocardial ischemia-reperfusion, and heart failure. Multiple antioxidant therapies (inhibition of oxidative stress producers, improvement of endogenous antioxidant capacity, and supplementation of exogenous antioxidants) and statins have been tested in order to counteract oxidative stress-induced cardiac damages. DNA: deoxyribonucleic acid; GSHPsx: glutathione peroxidase; H_2_O_2_: hydrogen peroxide; MAO: monoamine oxidase; NADPH: nicotinamide adenine dinucleotide phosphate hydrogen; NO: nitric oxide; NOS: nitric oxide synthase; O_2_^−^: superoxide anion; OH^−^: hydroxyl anion; ONOO^−^: peroxynitrite; ROS: reactive oxygen species; SOD: superoxide dismutase; XOR: xanthine oxidoreductase.

ROS represent important second messengers within the heart, since they are involved in multiple physiological processes including differentiation, proliferation, and excitation-contraction coupling (Figure 2). However, when the production of ROS exceeds the buffering capacity of the antioxidant defense systems in the heart, oxidative stress arises, resulting in cardiac dysfunction, ischemia-reperfusion injury, hypertrophy, cell death, and heart failure. Endogenous ROS in the heart are generated by mitochondria, xanthine oxidoreductase, uncoupled nitric oxide synthase, NADPH oxidase, cytochrome P450, and monoamine oxidases. ROS are also involved in the onset of some complications related to specific clinical settings, including chemotherapy-induced cardiotoxicity and POAF, as well as in the onset of diabetic cardiomyopathy, which represents a disorder of the heart in diabetic patients in the absence of other comorbidities related to diabetes. Multiple antioxidative stress therapies have been tested through different approaches: inhibition of oxidative stress producers, improvement of endogenous antioxidant capacity, and improvement of antioxidant capacity by supplementation of exogenous antioxidants. However, the results from these clinical trials suggest that although targeting oxidative stress is theoretically logical, the majority of the current strategies fail to improve patient outcomes and prognosis. The results obtained using drugs with anti-inflammatory, antioxidative stress and antidiabetic properties appear to be more promising. The improvement of experimental settings and knowledge about the pharmacokinetic of antioxidants, as well as the identification of more specific markers and the use of larger study cohorts, will lead to the identification of novel, more effective therapeutic approaches for heart diseases related to oxidative stress.

## Figures and Tables

**Table 1:** Potential sources of ROS in the heart. There are multiple sources of ROS in the heart, including those arising from NADPH oxidase, xanthine oxidoreductase, nitric oxide synthases, monoamine oxidases, mitochondria, and cytochrome P450. Their role in generation of oxidative stress, how their activity is modulated, and the specific mechanisms of action are also described. BH_2_: dihydrobiopterin; BH_4_: tetrahydrobiopterin; CYP2E1: cytochrome P450 2E1; eNOS: endothelial NOS; ETC: electron transport chain; iNOS: inducible NOS; I/R: ischemia-reperfusion; LV: left ventricular; MAO: monoamine oxidases; NADPH: nicotinamide adenine dinucleotide phosphate hydrogen; NO: nitric oxide. NOSs: nitric oxide synthases; Nox: NADPH oxidases; nNOS: neuronal NOS; POAF: postoperative atrial fibrillation; PPAR*α*: peroxisome proliferator-activated receptor alpha; ROS: reactive oxygen species; XDH: xanthine dehydrogenase; XO: xanthine oxidase; XOR: xanthine oxidoreductase.

NADPH oxidases (Nox)	(i) Nox catalyze the reduction of O_2_ to O_2_^−^ by using NADPH as electron donor. (ii) Nox activity increases in the failing heart and in angiotensin-II-induced cardiac hypertrophy. (iii) ROS produced by Nox can promote further ROS generation by other sources, such as XOR, and degrade BH_4_. (iv) ROS generated by the Nox family of NADPH oxidases may act as second messengers regulating cell growth and differentiation. (v) Suppression of Nox2 and Nox4 below physiological levels is able to exacerbate myocardial I/R injury, whereas a minimum level of ROS production by either Nox2 or Nox4 is essential for the activation of hypoxia-inducible factor-1*α* (HIF-1*α*) and inhibition of PPAR*α* during I/R. (vi) ROS specifically derived from the Nox2 NADPH oxidase give a relevant contribution to the development of cardiac remodeling associated with chemotherapy-induced cardiotoxicity.

Xanthine oxidoreductase (XOR)	(i) XDH and XO oxidate xanthine to uric acid promoting a flux of electrons to reduce NAD^+^ to NADH (XDH) or O_2_ to H_2_O_2_ and O_2_^−^ (XR). (ii) XDH/XO protein expression is increased in failing heart. (iii) XO inhibition reverses LV remodeling and improves LV function in rats.

Mitochondrial ROS	(i) ROS generation is related to the partial reduction of O_2_ to O_2_^−^ by complexes I and III of the ETC and to the protein p66^shc^. (ii) Mitochondrial oxidant modifications attenuate cardiac aging, protect from cardiac disease, and prevent left ventricular remodeling and failure in animal models. (iii) Complexes I and III are the best characterized enzyme complexes mediating ROS generation in the mitochondria and are responsible for the majority of mitochondrial ROS in cardiovascular physiology and disease. (iv) Mitochondrial ROS are also generated by reverse electron transport at mitochondrial complex I. (v) Mitochondrial ROS affect a broad range of cellular functions in the context of heart failure. (vi) The increased mitochondrial calpain-1 is associated with mitochondrial ROS generation in diabetic cardiomyopathy.

NOSs	(i) NOSs catalyze the production of NO and citrulline from oxygen and L-arginine. (ii) nNOS-derived NO may inhibit XOR activity, limiting myocardial oxidative stress and increasing NO availability within the myocardium. (iii) iNOS upregulation and overexpression induce cardiac apoptosis, fibrosis, hypertrophy, and dilatation in animal models. (iv) ROS generated by eNOS oxidize BH_4_ to BH_2_ and increase metalloproteinases activation. (v) In the presence of high-glucose concentration, eNOS becomes unstable and electrons become diverted to molecular oxygen rather than to L-arginine, resulting in O_2_^−^ formation and leading to eNOS uncoupling. (vi) Pressure overload triggers eNOS uncoupling, which in turn contributes to dilatory remodeling and cardiac dysfunction. (vii) O_2_^−^ from Nox may activate XOR and degrade BH_4_ leading to NOS uncoupling, as observed in diabetes and hypertension.

Monoamine oxidases (MAO)	(i) MAO expression and their ability to produce ROS increase with age and in age-associated chronic diseases. (ii) MAO activity is associated with an increased risk for POAF. (iii) MAO-dependent oxidative stress also contributes to mast cell degranulation and cardiac fibrosis, ultimately resulting in diastolic dysfunction in type 1 diabetes. (iv) MAO-A-induced oxidative stress triggers p53 activation and impairs lysosome function and acidification. (v) Genetic deletion of MAO-A is protective in I/R injury, pressure overload, and heart failure. (vi) Genetic deletion of MAO-B protects against oxidative stress, apoptosis, and ventricular dysfunction.

Cytochrome P450 oxidase	(i) CYP2E1 is among the most active CYPs in producing ROS. (ii) The expression level of CYP2E1 increases significantly in human heart tissues under ischemia. (iii) CYP2E1 is an important gene in the pathogenesis of dilated cardiomyopathy in animal models. (iv) Marked expression of CYP2E is associated with several cellular markers of oxidative stress, including products of lipid peroxidation, and with increased cardiomyocytes apoptosis both *in vitro* and *in vivo*.

p66^shc^	(i) p66^shc^ protein is a 66 kDa cytosolic protein encoded by the Shc gene that upon stress may translocate to mitochondria and accept electrons from cytochrome C resulting in the formation of H_2_O_2_. (ii) Studies carried out by comparing hearts from p66^shc^ knockout and wild-type mice have highlighted the cardioprotective effects elicited by p66^shc^ ablation, since this protects against I/R insults.
